# No evidence for cardiotoxicity of miltefosine^☆^

**DOI:** 10.1016/j.abd.2022.03.002

**Published:** 2022-05-30

**Authors:** Thomas P.C. Dorlo

**Affiliations:** a Department of Pharmacy & Pharmacology, Antoni van Leeuwenhoek Hospital/Netherlands Cancer Institute, Amsterdam, The Netherlands; b Department of Pharmacy, Uppsala University, Uppsala, Sweden

Dear Editor,

In a recent article, Barroso et al.^1^ reported on the comparison of cardiotoxicity between N-methyl glucamine antimoniate, better known as meglumine antimoniate, and miltefosine. As the authors indicate, antimonial compounds are notorious for their extensive toxicity, particularly cardiotoxicity, leading to pronounced prolongation of corrected QT interval (QTc) and risk of developing abnormal heart rhythms that can cause sudden death.

The only significant result the authors report is a decreased relative risk in QTc > 440 msec for meglumine antimoniate (*n*=38) compared to miltefosine (*n*=15) on day 7 of treatment. Looking at it longitudinally for the miltefosine-regimen, the proportion of patients with QTc > 440 msec appeared to increase in the first week of treatment, but thereafter completely disappeared: 1/15, 5/15, 0/15, 0/15, for day 0, 7, 14, and 21, respectively, while it steadily increased in the meglumine antimoniate-treated group, culminating to 35.3% of patients with an abnormal QTc. Instead of looking at relative risk at each of the measured time points, assessing longitudinal changes within treatment groups would potentially have been a more relevant way of analyzing and interpreting these data.

The authors suggest that the cardiotoxicity of miltefosine has been described before, but to the best of my knowledge, this is not the case. The phase 3 study^2^ to which the authors refer reports no abnormalities in electrocardiography, with a 14 msec increase in QTc from baseline during miltefosine. Such a clinically non-relevant increase in QTc is seen for many anti-infective drugs, also for those not exhibiting cardiotoxicity, probably due to recovery from infection and waning of fever, which can have a prolonging effect itself on QTc.^3^

From a pharmacokinetic angle, there is little rationale for a QTc prolongation only in the first treatment week. Miltefosine keeps accumulating during the 28-day treatment regimen, reaching a steady state in the last week of treatment.^4^ Maximal drug concentrations in the first week are < 50% of those expected in the fourth week of treatment. Moreover, no mechanisms of cardiotoxicity are known from pre-clinical and clinical pharmacological research on miltefosine. Recently, a dedicated study investigating the effect of miltefosine on QTc in 42 Bolivian mucocutaneous leishmaniasis patients was concluded. While results remain to be published, the updated FDA miltefosine label mentions no evidence of QTc prolongation or increases >20 msec from baseline were observed in this study.

The conclusion should be that there is no convincing evidence nor pharmacokinetic-pharmacodynamic rationale that miltefosine causes cardiotoxicity.

## Financial support

None declared.

## Author’ contributions

Thomas P.C. Dorlo: Preparation and writing of the manuscript; study conception and planning; approval of the final version of the manuscript.

## Conflicts of interest

None declared.
